# Sensor and Sensorless Fault Tolerant Control for Induction Motors Using a Wavelet Index

**DOI:** 10.3390/s120404031

**Published:** 2012-03-27

**Authors:** Khalaf Salloum Gaeid, Hew Wooi Ping, Mustafa Khalid, Ammar Masaoud

**Affiliations:** Department of Electrical Engineering, University of Malaya, 50603 Kuala Lumpur, Malaysia; E-Mails: wphew@um.edu.my (H.W.P.); mkm_imn@hotmail.com (M.K.); ammarshz@yahoo.com (A.M.)

**Keywords:** fault tolerant control, V/f, vector control, BMRAS, wavelet, induction motor, stator winding shorts, stator winding open, speed sensor

## Abstract

Fault Tolerant Control (FTC) systems are crucial in industry to ensure safe and reliable operation, especially of motor drives. This paper proposes the use of multiple controllers for a FTC system of an induction motor drive, selected based on a switching mechanism. The system switches between sensor vector control, sensorless vector control, closed-loop voltage by frequency (V/f) control and open loop V/f control. Vector control offers high performance, while V/f is a simple, low cost strategy with high speed and satisfactory performance. The faults dealt with are speed sensor failures, stator winding open circuits, shorts and minimum voltage faults. In the event of compound faults, a protection unit halts motor operation. The faults are detected using a wavelet index. For the sensorless vector control, a novel Boosted Model Reference Adaptive System (BMRAS) to estimate the motor speed is presented, which reduces tuning time. Both simulation results and experimental results with an induction motor drive show the scheme to be a fast and effective one for fault detection, while the control methods transition smoothly and ensure the effectiveness of the FTC system. The system is also shown to be flexible, reverting rapidly back to the dominant controller if the motor returns to a healthy state.

## 1. Introduction

Practical control systems are susceptible to component malfunctions which may cause significant performance degradation and even instability of the system. The past two decades have therefore seen considerable research on Fault Tolerant Control (FTC). FTC systems are designed to allow recovery from damage and system faults. When it comes to electrical drives used in safety critical applications or industrial processes where system faults may lead to enormous costs, FTC systems are crucial [1]. Stator, rotor and shaft faults together constitute up to 47% of recorded induction motor faults [2].

Fourier Transform (FT) techniques, such as those using high resolution frequency estimation [3] and signal demodulation [4] have been applied to fault detection. The drawback of FT techniques is that they provide information only of the frequency domain, not the time domain [5]. Also, Fourier Transform does not allow the use of current as a basis for fault detection, because the current through a faulty motor is non-stationary and contains minor transients [6]. Artificial intelligence techniques have also been proposed [7–9]. A very promising avenue in motor fault detection is wavelet-based. Wavelets provide both time and frequency domain information. Chow *et al.* [10] used a Gaussian-enveloped oscillation wavelet for fault detection, although they restricted their study to mechanical faults. A more extensive wavelet-based fault detection algorithm was used by Schmitt *et al.* [11], with open winding faults, unbalanced voltage and unbalanced stator resistance taken into consideration. No hardware implementation was presented, however. Most recently, detection of stator winding shorts was presented in [12]. The work focused only on one type of fault.

In this paper, a fault tolerant control strategy which deals with a wide range of induction motor faults is implemented. A vector control drive with an encoder is the dominant control scheme. In the event of an encoder fault, the system switches to sensorless vector control. If the stator winding is open circuited or shorted, a closed loop V/f controller takes over. If a minimum voltage fault occurs, the system goes to open loop V/f control. Even further deterioration activates a protection circuit which halts the motor. Faults are detected using a wavelet index.

The four different controllers ensure the effectiveness and availability of the control scheme. The wavelet index is shown to be an excellent fault indicator. Additionally, the system has the ability to revert back to the dominant controller if the motor resumes normal operation, thus ensuring its availability at all times. Moreover, the protection circuit requires no extra hardware, thus reducing the cost of the drive. Additionally, the sensorless vector control features a novel Boosted Model Reference Adaptive System (BMRAS) to estimate the speed that eliminates the need for a PI controller and thus of much tuning. The fault tolerant algorithm was executed initially through Matlab/Simulink and then was verified experimentally.

This paper is organized as follows. Section 2 describes the motor control strategies used in this work. The BMRAS controller is presented in Section 3. Section 4 explains the wavelet transform. The fault tolerant control strategy is described in Section 5. The experimental results are presented in Section 6. Finally, concluding remarks are given in Section 7.

## 2. Control Strategies of the Induction Motor

### 2.1. Sensor Vector Control

Vector control decouples flux and torque currents so as to linearly control the output torque of a nonlinear induction motor. The three phases of voltage and current are transformed to two-phase dq axes. The dq frame rotates synchronously with the rotor flux space vector. The expression for torque in an induction motor is [13]:

(1) $$T_{e}=\frac{3}{2}p\frac{L_{m}}{L_{r}}\left(\Phi_{\text{rd}}i_{\text{sq}}-\Phi_{\text{rq}}i_{\text{sd}}\right)$$

**Figure 1.**
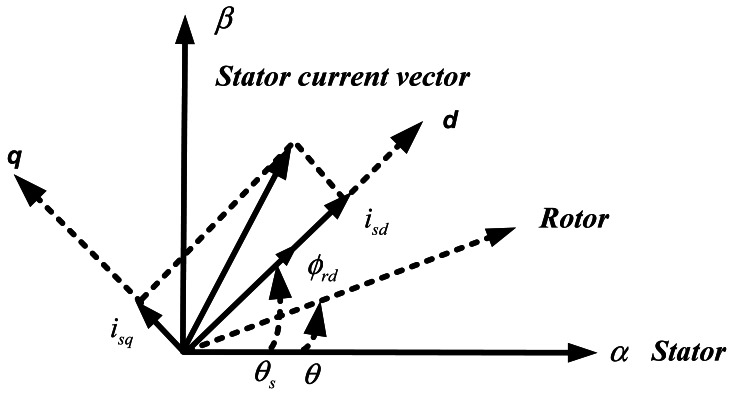
Reference frame for vector control.

According to the orientation of Figure 1, Φ*_rq_* becomes zero. The new expression becomes:

(2) $$T_{e}=\frac{3}{2}p\frac{L_{m}}{L_{r}}\left(\Phi_{\text{rd}}i_{\text{sq}}\right)$$

where *L_m_, L_r_, p, Φ_rd_, Φ_rq_, i_sd_, i_sq_* and *T_e_* are mutual inductance, rotor inductance, pole pairs, direct rotor flux and quadratic rotor components, direct stator current component, quadratic stator current component and electromangnatic torque, respectively.

As is clear from Equation (2), the motor torque can be controlled by controlling the quadrature component of stator current *i_sq_*.

Vector control with a sensor is the dominant controller in this work, due to its straightforward implementation. The following calculations are carried out in the vector control according to the Park transformation:

(3) $$\left[\begin{array}{l} i_{\text{qs}}\\ i_{\text{ds}} \end{array}\right]=\left[\begin{array}{ll} \;\cos\varphi & \sin\varphi \\ -\sin\varphi & \cos\varphi \end{array}\right]\;\left[\begin{array}{l} i_{Q}\\ i_{D} \end{array}\right]$$

**Figure 2.**
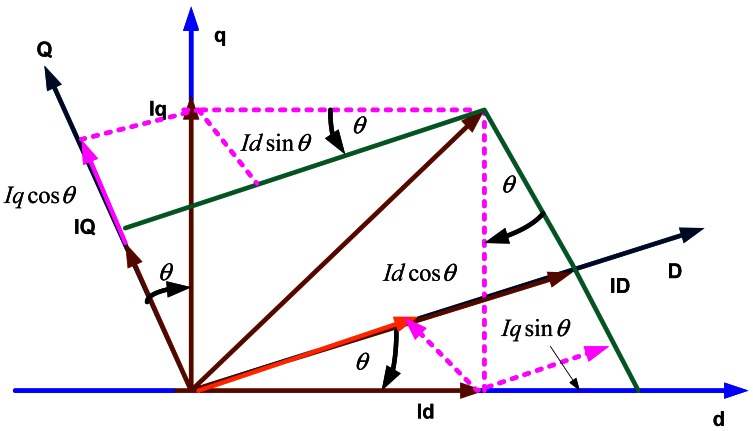
Park transformation principle.

This operation can be illustrated in Figure 2.

dq to abc transformation is:

(4) $$\left[\begin{array}{l} i_{\text{as}}\\ i_{\text{bs}}\\ i_{\text{cs}} \end{array}\right]=\left[\begin{array}{ll} \;1 & \;0\\ -0.5 & -\sqrt{3}/2\\ -0.5 & -\sqrt{3}/2 \end{array}\right]\;\left[\begin{array}{l} i_{\text{ds}}\\ i_{\text{qs}} \end{array}\right]$$

Therefore, the rotor flux and the torque can be independently controlled to obtain a linear current/torque relationship through the stator current in the *dq*-axis.

**Figure 3.**
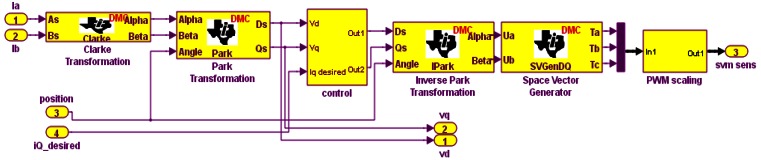
Simulink implementation of sensor vector control.

The Simulink model is shown in Figure 3.

### 2.2. Sensorless Vector Control

**Figure 4.**
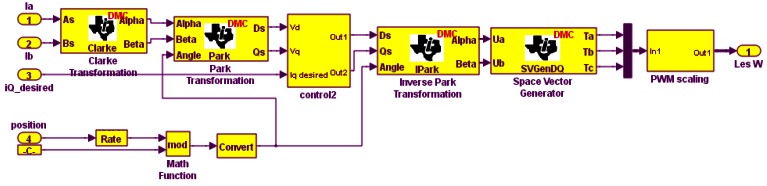
Simulink implementation of sensorless vector controller.

The encoder used for position and speed measurement may lead to problems. Faults such as loss of output information, offset, disturbances, measure deviation and channel mismatch may occur [14]. Sensorless vector control of induction motor drives estimates position using an observer and eliminates the need for the speed sensor. It reduces hardware complexity, size, maintenance and ultimately cost. It also eliminates direct sensor wiring and has been shown to have better noise immunity and increased reliability [15]. The Simulink implementation of sensorless vector control is shown in Figure 4.

### 2.3. Volt to Frequency (V/f) Control

The V/f control is one of the most popular control techniques due to the following reasons:

1. It is a simple algorithm
2. There is no need of current sensors
3. There is no requirement of speed measurement

The following equations can explain the principle of V/f:

(5) $$\hat{V}\approx j\omega \hat{\Lambda }$$

where *ω* and Λ̂ are the phasors of stator voltage and stator flux respectively:

(6) $$\left|\hat{V}|\approx |j\omega \hat{\Lambda }\right|$$

(7) V≈2πfΛ

(8) $$\Lambda =\frac{1}{2\pi f}V\;\text{or}\Lambda =\frac{1}{2\pi }\frac{V}{f}$$

The stator flux remains constant if the ratio V/F remains constant despite the change in the frequency.

The stator flux in an induction motor is proportional to the ratio of applied voltage and supply frequency. Varying the frequency changes the speed. With the voltage to frequency maintained at the same ratio, flux and torque can be kept constant throughout the speed range. The speed is adjusted by varying frequency (*f*), maintaining V/f constant to avoid flux saturation as is shown in the following equations:

(9) $$E_{\text{airgap}}=\text{kf}\phi_{\text{airgap}}$$

For constant air gap flux (*ϕ_airgap_*):

(10) $$E_{\text{airgap}}/f\approx v/f$$

It is a much simpler control strategy than vector control and does not require high performance digital processing [16], which makes it suitable as a backup control strategy in the event of faults. While it is generally implemented in open loop, a closed loop approach is also adopted for higher accuracy of the speed response. A PI controller regulates the slip speed of the motor to keep the motor speed at its set value.

## 3. Boosted Model Reference Adaptive System (BMRAS)

Model Reference Adaptive Systems (MRAS) are used to estimate quantities using a reference model and an adaptive model. The difference between the outputs of the two models drives an adaptive mechanism that provides the quantity that is to be estimated. Conventional MRAS use a simple fixed gain linear PI controller to generate the estimated rotor speed. This PI controller consumes time for tuning. In this work, the PI controller is replaced with a ‘booster’, which cuts down on tuning time while providing a good response. The booster is constructed using a rate limiter and zero order hold.

Taking the system shown in [17], the reference model can be expressed in the following equations:

(11) $$p\lambda_{\text{dr}}=L_{r}/L_{m}(v_{\text{ds}}-R_{s}i_{\text{ds}}-\sigma L_{s}di_{\text{ds}}/dt)$$

(12) $$p\lambda_{\text{qr}}=L_{r}/L_{m}(v_{\text{qs}}-R_{s}i_{\text{qs}}-\sigma L_{s}di_{\text{qs}}/dt)$$

The adaptive model can be expressed in the following equations:

(13) $$p\lambda_{\text{qr}}^{\prime }=(L_{m}/T_{r})i_{\text{qs}}-(L_{m}/T_{r})\lambda_{\text{qr}}^{\prime }+\omega_{r}\prime \lambda_{\text{dr}}^{\prime }$$

(14) $$p\lambda_{\text{dr}}^{\prime }=(L_{m}/T_{r})i_{\text{ds}}-(L_{m}/T_{r})\lambda_{\text{dr}}^{\prime }-\omega_{r}\prime \lambda_{\text{qr}}^{\prime }$$

(15) $$ɛ=\lambda_{\text{qr}}\lambda_{\text{dr}}^{\prime }-\lambda_{\text{dr}}\lambda_{\text{qr}}^{\prime }$$

where *R_s_, L_s_, V_ds_, V_qs_, T_r_, w_r_* are stator resistance, stator inductance, direct component of stator voltage, quadratic component of stator voltage, rotor time constant and rotor speed, respectively.

**Figure 5.**
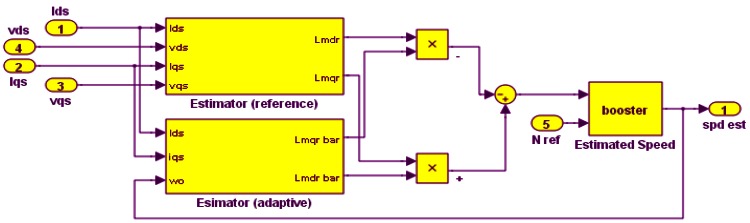
Simulink implementation BMRAS.

The error between the reference and adaptive outputs, along with the reference speed (*N_ref_*) is passed to the booster block shown in Figure 5.

The initial condition of both signals is kept to zero. The rate limiter restricts the change of the signal passed to it by limiting the slope. The upper limit is called the rising slew parameter (*δ*) and the lower limit is the falling slew parameter (*γ*). The output of the rate limiter is calculated as follows:

(16) $$O_{o/p}(i)=\nabla t.\delta +N(t-1)$$

(17) $$O_{o/p}(i)=\nabla t.\gamma +N(t-1)$$

(18) $$O_{o/p}(i)=N(i)$$

where *N* refers to the input to the rate limiter. The output is passed to a Zero Order Hold (ZOH) to generate continuous time input by holding each sample value constant over one sample period. The ZOH also acts as a hypothetical filter that gives a piece-wise signal as is demonstrated by the following equation:

(19) $$O_{{\text{ZOH}}_{o/p}}(t)=\sum_{n=-\infty }^{\infty }N_{\text{in}}[n].\text{rect}(\frac{t-\text{nT}}{T}-\frac{1}{2})$$

Finally, the estimated speed is calculated as follows:

(20) $$\text{spd est}(i)=N_{\text{ref}}(i)-\omega_{\text{Booster}}(i)$$

**Figure 6.**
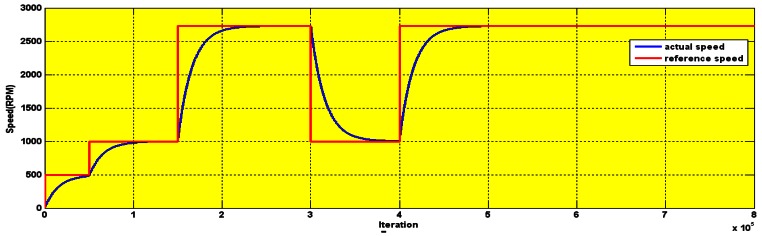
Simulation results of the speed tracking by the BMRAS.

**Figure 7.**
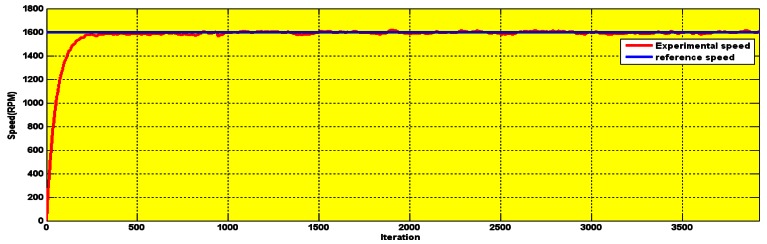
Experimental results of the speed tracking by the BMRAS.

The BMRAS was tested in both simulation and experiments (The experimental setup is described in Section 6). Figure 6 shows good tracking by the BMRAS of low speeds, high speeds and step changes in speed. There is no steady state error. The experimental results up to 1,600 rpm also show fast settling time and low steady state error (less than 30 rpm), as is seen in Figure 7.

The Figure 6 shows the full speed simulation with long operation period. The serial communication interface output of the experimental result for 3.5 s is shown in Figure7.

According to the computer simulation and experimental results shown above, the system shows fast response with higher accuracy than the conventional MRAS in the literature [18].

## 4. Wavelet Index

A wavelet is an orthogonal function that can be applied to a finite group of data [19]. While Fourier analysis techniques have been used extensively for induction motor fault diagnosis, they require large amounts of data [5]. Also, Fourier techniques inform us only about frequency components of signals, while wavelet transforms provide both time and frequency information. They are therefore more comprehensive and have wider ranging applications. Wavelet coefficients, at a first level of decomposition, are obtained from a signal by applying a mother wavelet, which represents a family of functions that need to satisfy a number of criteria. The mother wavelet, denoted by *ψ(t)*, must have a zero mean as shown in Equation (21):

(21) ∫ψ(ω)=0

It must also have a square norm of one, as is seen in Euqation (22):

(22) $${\left|\psi (\omega )\right|}^{2}=1$$

A general equation of the mother wavelet, shown in Equation (23), that shows the family of wavelets it represents, can be shown by adding a translation and scaling factor *a* and *b*, respectively:

(23) $$\omega_{a,b}(t)={\left|a\right|}^{-1/2}\psi (\frac{t-b}{a})$$

Wavelet coefficients are obtained using a low pass filter to obtain what is called an ‘approximation’ signal, while a high pass filter provides ‘details’. The approximation signal is progressively decomposed into further approximations and details, till a desired level of decompositions is obtained [11]. In this work, changes in the waveform of stator current are used as the basis for detecting faults. The current signal is passed through the wavelet transform. For every detail obtained from the high pass filter, the energy is calculated by adding the squared coefficients of the details and the final approximation. The maximum energy serves as the most effective piece of information to determine the wavelet index. The index is calculated according to Equation (24):

(24) $$W_{\text{indx}}=\text{abs}(\text{energy of selected level})/\text{average}(\text{energy}(\text{Ia}))$$

The energy is calculated according to Equation (25):

(25) $$\text{energy of selected level}=\int {d8}^{2}\text{dt}$$

where d8 refers to the 8th decomposition detailed frequency and information about the stator current status obtained from the high pass filter.

**Figure 8.**
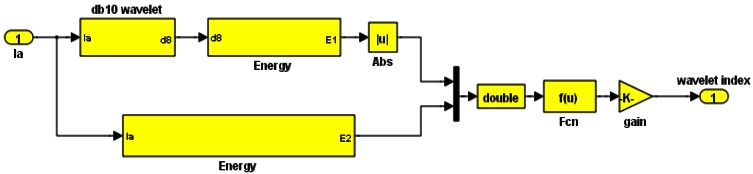
Simulink wavelet index implementation.

A Daubechies wavelet (db10) is the mother wavelet function using which the wavelet index is generated. The Simulink implementation is shown in Figure 8.

The wavelet decomposition levels used in Equation (24) was performed according to the following criteria:

(26) $$W_{\text{decomp}}=\frac{\log(f_{s}/f)}{\log(2)}\pm 1$$

where *f_s_* is the sampling frequency (20 kHz) and *f* is source frequency.

The optimal levels of decomposition are gauged through the optimum mother wavelet. The Shannon entropy orientates the route in the selection of this optimal level by determining the entropy of each original (parent) subspace of the (DWT) and also views it in comparison to its new (children) subspace.

## 5. Fault Tolerant Control

**Figure 9.**
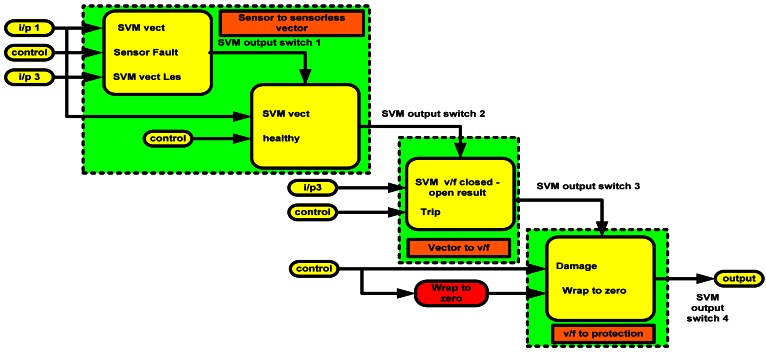
Switching mechanism between the controllers.

**Figure 10.**
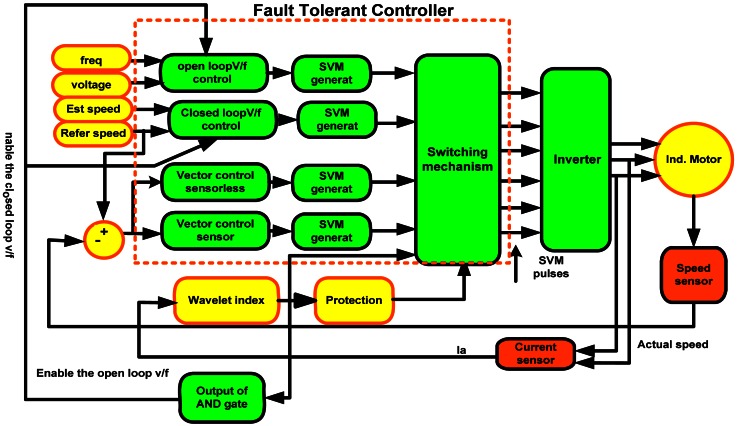
Fault tolerant control algorithm.

Fault tolerant control is indispensable, especially taking into consideration the formidable costs of unplanned stops in industrial system operations. The mechanism to switch between controllers in the event of fault and the overall fault tolerant control scheme used in this work is shown in Figures 9 and 10, respectively.

In the Figure 9, the trip is a binary indication of fault and is either 0 or 1. The control signal determines the type of fault and SVM seen in the figure. Figure 10 shows the flow of the SVM signal.

In this work, four control strategies are used. In normal operation, sensor vector control runs the drive. When an encoder fault occurs, sensorless vector control takes over. An open circuit in the stator winding or a short reverts the system to closed loop V/f control. V/F controlled drives are very reliable due to the restriction to low dynamic performance and the absence of closed loop control, while a minimum voltage fault enables open loop V/f control to maintain acceptable level of operation due to the degradation of the system performance and the difficulties of keeping good performance with the closed loop.

**Figure 11.**
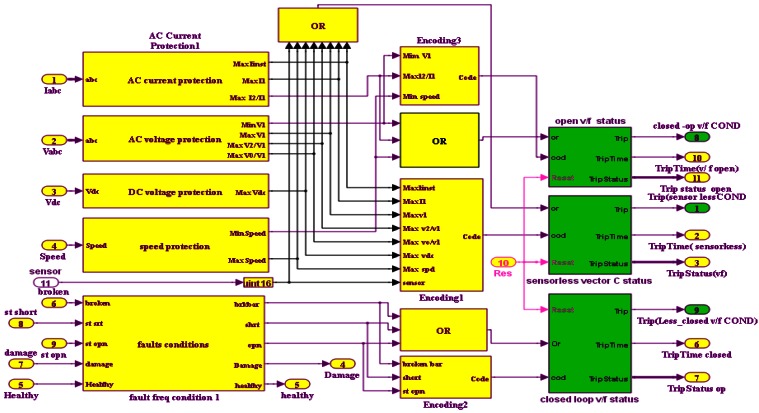
Simulink model of FTC system.

If a slight noise is wrongly interpreted as a fault, the system quickly reverts back to sensor vector control. Finally, the protection circuit is enabled in the event that two or more faults occur at once. Digital motor control blocks (DMC) are used to simulate the proposed algorithm due to their easy compilation from Simulink/Matlab to C++ or C through the Texas Instruments F28335 DSP. The Simulink model is shown in Figure 11.

## 6. Experimental Results

Experimental setup of the induction motor drive is based on the TMS320F28335 DSP. The induction motor parameters are listed in Table 1.

**Figure 12.**
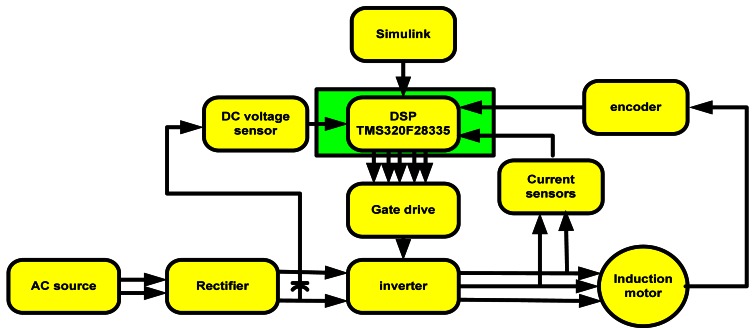
Hardware implementation scheme.

**Figure 13.**
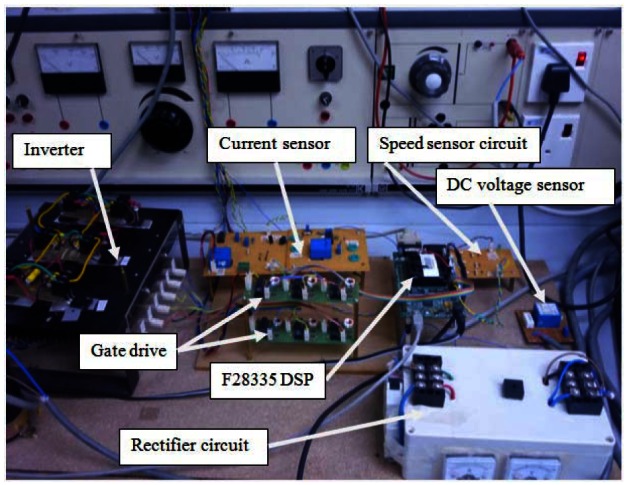
Circuits of the induction motor drive.

**Figure 14.**
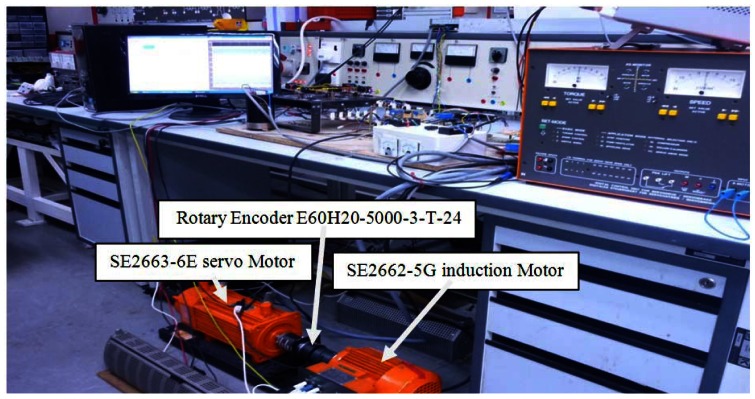
Induction motor setup.

The hardware scheme is depicted in Figure 12 and shown in picture in Figures 13 and 14.

### 6.1. Performance under Healthy Operation

**Figure 15.**
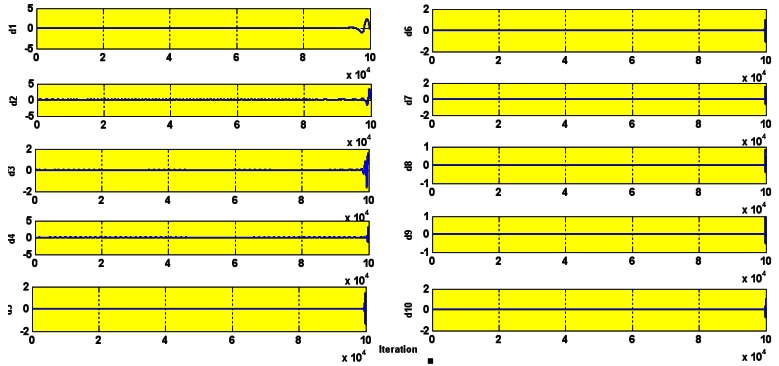
Wavelet decomposition in healthy motor.

**Figure 16.**
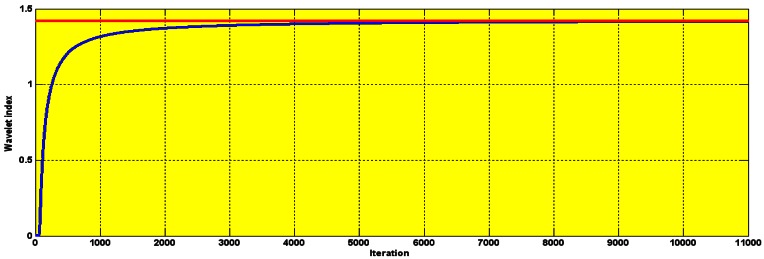
Experimental (red) and simulation comparison of the healthy I.M wavelet index.

The wavelet decompositions of stator current in the healthy induction motor are shown in Figure 15. The lack of any heavy perturbation shows that the motor is healthy (faultless). The small perturbation is negligible and is simply because of the high sensitivity of the wavelet which we actually use to our advantage. The experimental and simulation wavelet indices are compared in Figure16.

The amplitude of the wavelet index for healthy operation, as seen in Figure 16, is 1.4. The crossing of this threshold is an indication of a fault.

**Figure 17.**
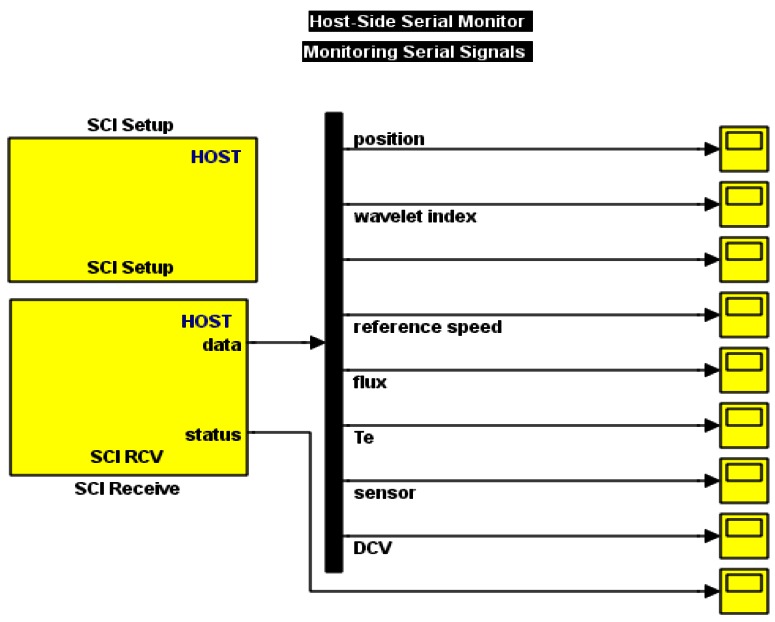
Serial communication Interface to show the experimental output.

The monitoring of the system parameters can be obtained through a serial communication cable between the DSP and the PC using SCI transmit and receive blocks as is shown in Figure 17.

To demonstrate the effectiveness of the fault tolerant algorithm, three faults are investigated: Short and open circuits in the stator winding and sensor faults. At each fault, the appropriate wavelet index is calculated, as is demonstrated in the following sections.

### 6.2. Stator Winding Short

**Figure 18.**
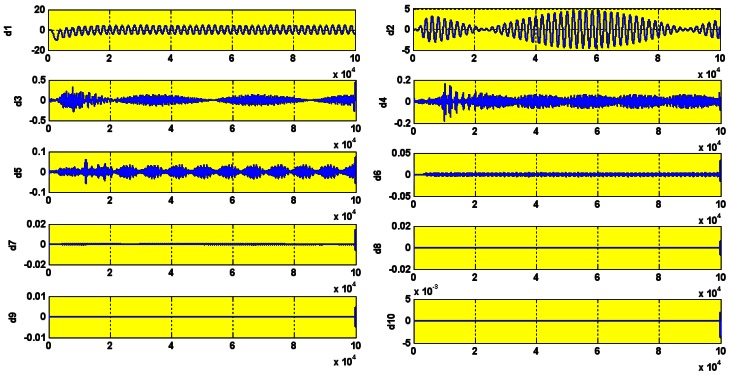
Wavelet decomposition.

To simulate this fault, the stator resistance was decreased 10 times in steps of 0.1 Ω. The motor has a delta connection. The variable resistance serves to reduce the stator resistance according to the equation for equivalent resistance of two parallel resistors. For each shunt resistance value, the mean wavelet index is calculated. The wavelet decomposition details are shown in Figure 18.

Experimental responses of the drive at 450 rpm, 900 rpm and 1,600 rpm were obtained with this fault. At each speed, the wavelet index was recorded and compared to the simulation results as is detailed below:

#### 6.2.1. At 450 rpm

The first test was with a speed of 450 rpm. The wavelet index comparison between experimental and simulation results at this speed is listed in Table 2. It shows the amplitude of the wavelet index increases to 1.5 due to the winding short introduced.

#### 6.2.2. At 900 rpm

The second test is with a maximum speed of 900 rpm. As its clear from Table 2, the wavelet index increases to 1.8 for the winding short at 900 rpm.

#### 6.2.3. For 1,600 rpm

The wavelet index lies between 1.8 and 2 for a stator winding short at 1,600 rpm as is seen in Table 2. The data shows a slight difference between the wavelet indices for the different speeds. The reason for that is the distortion in the stator current waveform in the experimental test.

### 6.3. Stator Winding Open Circuit

**Figure 19.**
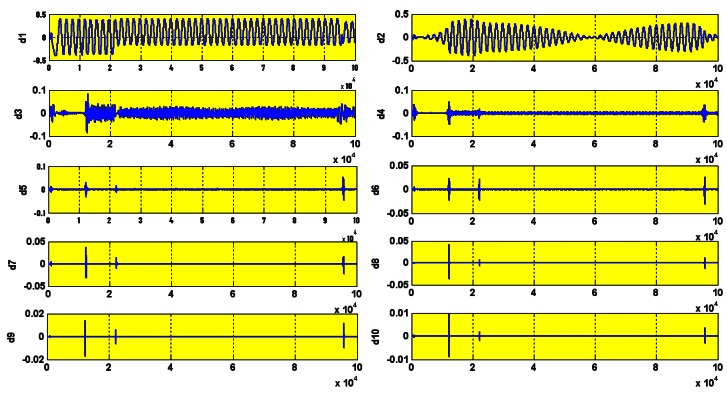
Wavelet decomposition Rs = 200 Ω.

To introduce the open circuit fault, the stator resistance was increased 10 times the original (20 Ω) in steps of 2 Ω. The wavelet decomposition of the faulty stator current is shown in Figure 19.

**Figure 20.**
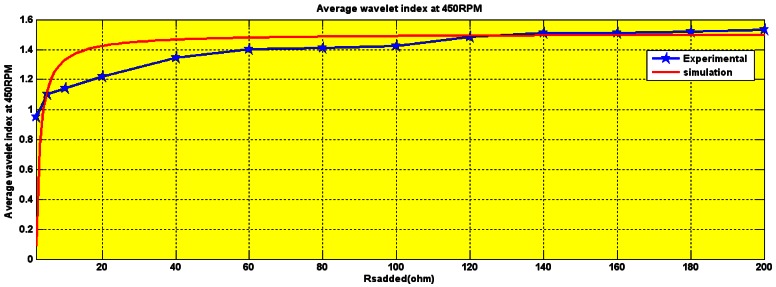
Experimental and simulation Wavelet index Ohm at 450 rpm.

**Figure 21.**
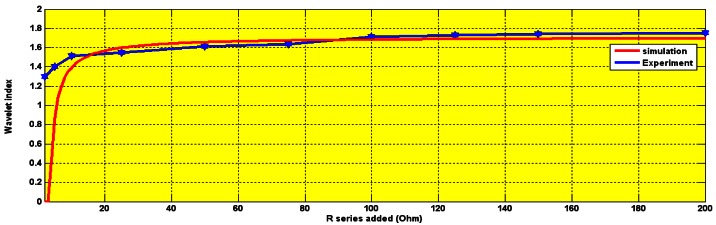
Experimental and simulation wavelet index for series Rs (2–200) Ohm at 900 rpm.

**Figure 22.**
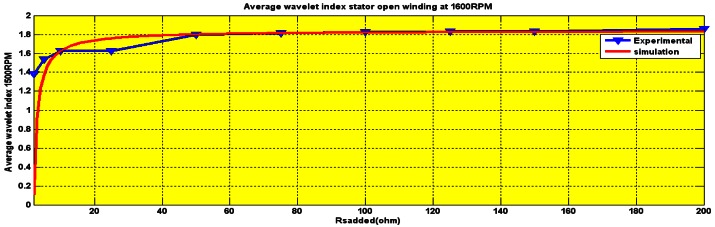
Wavelet index for series Rs (2–200) Ohm at 1,600 rpm.

The wavelet index was recorded to be 1.5 for 450 rpm, between 1.2 and 1.6 for 900 rpm and 1.8 at 1,600 rpm as is shown in Figures 20, 21 and 22, respectively.

### 6.4. Encoder Faults

**Figure 23.**
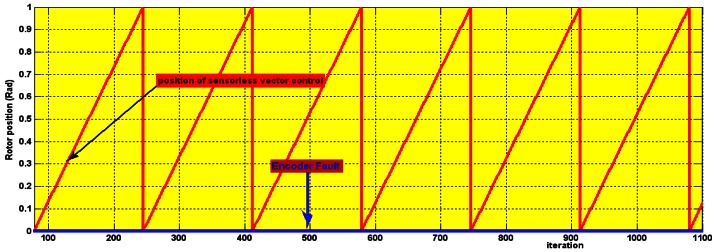
Experimental rotor position: Complete sensor failure.

Two types of speed sensor (encoder) faults are presented in this work. The first is complete speed sensor failure as is depicted in Figure 23. To introduce complete speed sensor failure the cables of the encoder channels A, B and index I were disconnected. The blue line is the encoder output (zero) when it fails. The red line is the rotor position estimated with the BMRAS, as the system switches to sensorless operation when the sensor fault is detected.

**Figure 24.**
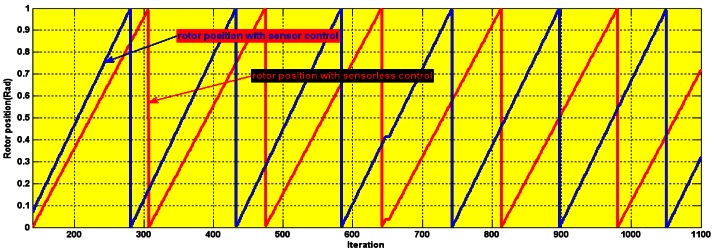
Experimental rotor position with partial sensing error.

The second type of sensor fault was a partial sensing error in the position, which was created by introducing noise in the encoder LED. The encoder output in Figure 24 depicts this fault.

**Figure 25.**
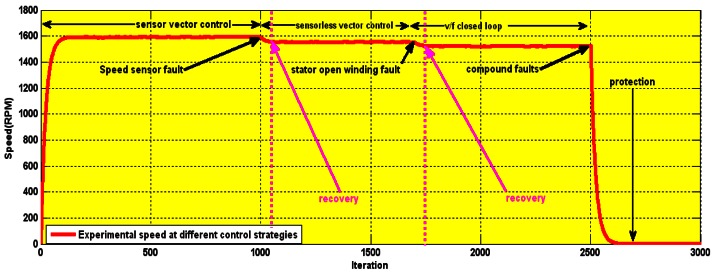
Experimental speed transition with different controllers.

The fault tolerant algorithm was tested with these faults at different speeds. Before starting the induction motor, the cables of the encoder channels were disconnected. As is seen in Figure 25, the encoder fault is introduced at the 1000th iteration (3 s), at which point the system switches from sensor vector control to sensorless vector control. At 5 s a stator short winding fault is introduced and the system switches to closed loop V/f control. At 10 s, a compound fault (both stator winding open and short circuits simultaneously) is introduced which activates the protection unit and brings the motor to a halt. The protection unit is part of the software program and requires no extra hardware.

The recovery from a fault occurs rapidly and the transition from one control scheme to the other is seen to be smooth. The performance does not degrade considerably even as the control strategy changes.

**Figure 26.**
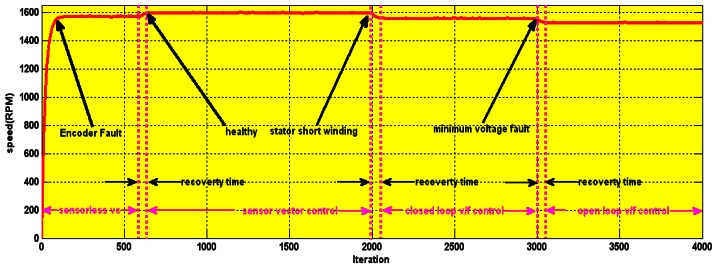
Control system recovery.

**Figure 27.**
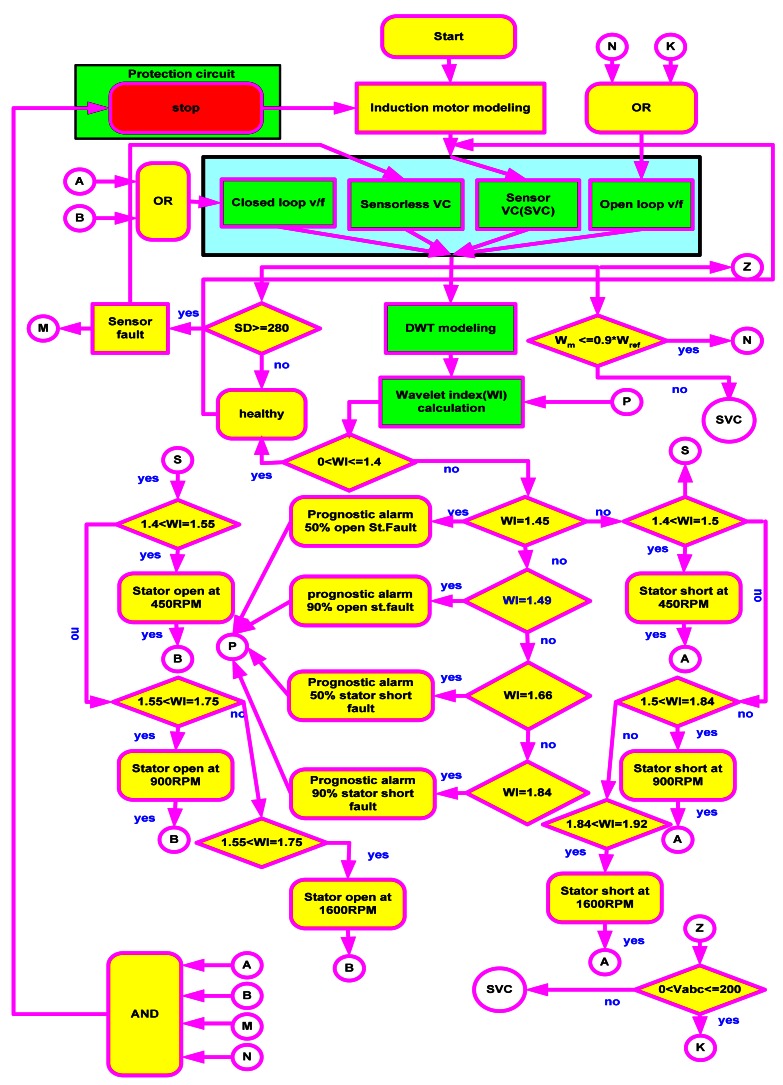
Expanded flow chart of the work.

The flexibility of the control strategy is depicted in Figure 26. The operation is started with an encoder fault. At the 550th iteration (1.5 s), the system returns to a healthy state. The system reverts back to sensor vector control with minimal recovery time. When a minimum voltage fault occurs at the 3,000th iteration, open loop V/f takes over. The general flow chart of the wavelet based fault tolerant control algorithm can be seen in Figure 27 (some parts of the flowchart are not included in this paper).

## 7. Conclusions

A fault tolerant control system incorporating (sensor and sensorless) vector control and (closed loop and open loop) V/f control has been presented. The wavelet index used for fault detection has been shown to be both fast and effective. The index detected complete sensor failures, partial sensor errors, stator winding shorts and open circuits and compound faults. The transitions from one controller to the other were both quick and smooth. The threshold of the WI is set according to the amplitude of the stator current, which differs for every fault.

The Boosted Model Reference Adaptive System (BMRAS) used in sensorless vector control was shown to be effective for rotor speed estimation. It saved time otherwise consumed in tuning the conventional PI controller, while maintaining excellent performance.

The system has been shown to be flexible, in that if a fault is removed and the system returns to a healthy state, the drive reverts back to the dominant sensor vector control. The protection unit was implemented successfully, not requiring additional hardware and thus saving cost.

Future work may consider adding strategies such as Direct Torque Control (DTC) to the control scheme. Additionally, a thorough analysis of the switching mechanism, such as time delays, would be useful. The inclusion of prognostic mechanisms, for an early prediction of faults before they occur, is also a very good prospect.

## Figures and Tables

**Table 1.** Induction motor parameters.

**Motor spec.**	**Value**
Power	1 kW
Current	2.5 A
Voltage (delta)	400 V
Rated Speed	2,780 rpm
No. of poles	2
Moment of Inertia	2.4e−4 kgm^2^
Stator Resistance	20.9 ohm
Rotor Resistance	19.5 ohm
Stator Inductance	50e−3 Henry
Rotor Inductance	50e−3 Henry

**Table 2.** Wavelet index (WI) for shunt Rs (0.1–2) for different speeds.

**R_sh_(Ω)**	**WI at 450 rpm**		**WI at 900 rpm**		**WI at 900 rpm**	

	**Exp.**	**Sim.**	**Exp.**	**Sim**	**Exp.**	**Sim.**
0.10	1.10	1.00	1.78	1.76	1.80	1.70
0.20	1.40	1.40	1.80	1.80	1.81	1.72
0.40	1.43	1.42	1.80	1.80	1.85	1.76
0.80	1.43	1.42	1.82	1.80	1.90	1.88
1.60	1.50	1.50	1.84	1.86	1.92	1.91
1.80	1.50	1.50	1.84	1.88	1.95	1.93
2.00	1.50	1.50	1.84	1.90	2.00	1.96

## References

[1] Gaeid K.S., Ping H.W. (2011). Fault tolerant control of induction motor. Mod. Appl. Sci..

[2] Cusidó J., Romeral L., Ortega J.A., Garcia A., Riba J. (2011). Signal injection as a fault detection technique. Sensors.

[3] Kia S.H., Henao H., Capolino G.A. (2007). A high-resolution frequency estimation method for three-phase induction machine fault detection. IEEE Trans. Ind. Electron..

[4] Stefani A., Filippetti F., Bellini A. diagnosis of induction machines in time-varying conditions.

[5] Ebrahim E.A., Hammad N. Fault analysis of current-controlled pwm-inverter fed induction-motor drives.

[6] Zanardelli W.G., Strangas E.G., Khalil H.K. (2002). Comparison of wavelet-based methods for the prognosis of failures in electric motors. Power Electronics in Transportation.

[7] Fiippetti F., Vas P. Recent developments of induction motor drives fault diagnosis using AI techniques.

[8] Ye Z., Wu B., Sadeghian A.R. Induction motor mechanical fault online diagnosis with the application of artificial neural network.

[9] Li B., Chow M.-Y., Tipsuwan Y., Hung J.C. (2000). Neural-network-based motor rolling bearing fault diagnosis. IEEE Trans. Ind. Electron..

[10] Chow T.W.S., Shi H. (2004). Induction machine fault diagnostic analysis with wavelet technique. IEEE Trans. Ind. Electron..

[11] Schmitt P.I., Morales A. (2010). Applications of wavelets in induction machine fault detection. Ingeniare. Rev. Chil. Ing..

[12] Bossio G.R., de Angelo C.H., de la Barrera P.M., Solsona J.A., Garcia G.O., Valla M.I. Stator winding fault detection in induction motor drives using signal injection.

[13] Boulghasoul Z., Elbacha A., Elwarraki E., Yousfi D. Combined vector control and direct torque control an experimental review and evaluation.

[14] Bourogaoui M., Berriri H., Ben Attia-Sethom H., Slama-Belkhodja I. Wavelets and parity equations methods comparison for faulty encoder detection in PMSM drives.

[15] Zaky M.S. (2012). Stability analysis of speed and stator resistance estimators for sensorless induction motor drives. IEEE Trans. Ind. Electron..

[16] Itoh J.I., Nomura N., Ohsawa H. A comparison between V/f control and position-sensorless vector control for the permanent magnet synchronous motor.

[17] Gadoue S.M., Giaouris D., Finch J.W. (2010). MRAS sensorless vector control of an induction motor using new sliding-mode and fuzzy-logic adaptation mechanisms. IEEE Trans. Energy Convers..

[18] Vasic V., Vukosavic S. (2001). Robust MRAS-based algorithm for stator resistance and rotor speed identification. IEEE Power Eng. Rev..

[19] Edwards T. (1991). Discrete Wavelet Transforms: Theory and Implementation.

